# In Honour of Em. o. Univ.-Prof. Mag. pharm. Dr. Wolfgang Kubelka on the Occasion of his 80^th^ Birthday

**DOI:** 10.3797/scipharm.ed-15-01

**Published:** 2014-11-22

Wolfgang Kubelka was born on February 18^th^, 1935 in Vienna. His scientific career started even before his graduation with a Master’s Degree in Pharmacy at the Institute of Pharmacognosy at the University of Vienna. From the beginning, he dedicated his scientific efforts to medicinal plants. In 1965, he finished his Ph.D. thesis dealing with cardiac glycosides of *Convallaria majalis*.

He had the chance to gain more experience in the field of natural products chemistry during his research stays in the laboratories of outstanding experts like the professors Reichstein (Basel), Tschesche (Bonn), Beal, and Doskotch (Ohio State University) during 1965–1971.

Convallaria accompanied the scientific work of Prof. Kubelka through all these years, resulting in the Habilitation for Pharmacognosy in 1973. He is author of more than 100 publications dealing, for example, with the isolation and characterization of plant constituents, development of analytical methods suitable for the proof of quality of herbal substances and herbal preparations, biosynthesis of plant constituents, chemotaxonomy, and ethnopharmacology. He always took his responsibility for the education of students of pharmacy very seriously and introduced, for example, pharmacobotanical field excursions into the curriculum.

From 1983 to 2003, Prof. Kubelka headed the Institute of Pharmacognosy at the University of Vienna. His dedication to the development of sciences at the University resulted in his election as dean for the faculty of natural sciences for several years.

Beside this scientific career, Prof. Kubelka always tried to link the sciences with practical implementation. His initiative was essential for the foundation of the Austrian Pharmaceutical Society (ÖPhG 1979) and the Austrian Society for Phytotherapy (ÖGPhyt 1991). Although being emeritus professor since 2003, he is still active in the training of pharmacists and physicians, in organizing congresses, and among other topics, in being chief editor of several journals. His expertise is also still acknowledged in the Quality Drafting Group of the Committee on Herbal Medicinal Products at the European Medicines Agency.

Since 1985, Prof. Kubelka has been the editor-in-chief of Scientia Pharmaceutica. During these years, he guided this journal from the printed version to the online edition with free access to full texts. The editorial board of Scientia Pharmaceutica congratulates Prof. Kubelka on the occasion of his 80^th^ birthday with the best wishes for his future.

*Ad multos annos! The Editors*

